# Supplementary material for: When Corona Infested Everything—A Qualitative Interview Study Exploring the Impact of COVID-19 Mitigation Measures on School Life from the Perspectives of English Secondary School Staff and Students
Source: Int J Environ Res Public Health. 2025 Jun 10;22(6):915. doi: 10.3390/ijerph22060915 (PMC12193610; doi:10.3390/ijerph22060915)
Supplement: Supplementary file 1 [file ijerph-22-00915-s001.zip › ijerph-3592739-supplementary.pdf]

Supplementary material

File S1. Interview guides

University of BRISTOL

CoMMinS

COVID-19 Mapping and Mitigation in Schools

School life during COVID 19

TOPIC GUIDE FOR SECONDARY SCHOOL STUDENTS

1. **Setting the scene**

Please can you tell me a little bit about yourself and your family?

What area do you live in? Who lives at home with you? Brothers and sisters?

Please can you tell me about your friends at school? What about activities outside school?

2. **Impact of COVID-19 on you and your family**

Could you describe how you have been feeling since going back to school in September?

How have things been at home?

3. Impact of COVID19 on being at school

What has been happening at school to try and keep everyone safe?

How do you feel about this (too little, too much, any anxieties?)

If young person talks about concerns and anxieties – Who do you talk to about your worries?

4. **Views and attitudes on mitigation measures**

    - **Social distancing**

    (changing room layouts, layout of school, limiting number of children in school, a mix of online and face to face teaching, wearing of face coverings)

How easy / difficult is it for you to follow social distancing in schools?

What could stops you doing this? (e.g. built environment/classroom space, fighting/bullying, peer pressure)

What helps you do this? (additional classroom space / teachers, markers on the ground, verbal and written information)

What times in the day is it easier/more difficult for you to social distance from others? (commute to school, arriving/leaving school, break and lunch times, during lessons, moving between lessons, PE lessons, in bathrooms, after school, weekends)

Do you think social distancing measures affect your learning and social life ?

Do you think social distancing measures are having an impact your mental wellbeing?

- **Hand and other hygiene measures**

How have you found following hand washing advice so far?

Do you have any concerns about possible negative impact of extra hand-washing?

How important do you think hand washing is at school (re COVID19)? What are you being asked to do to encourage frequency and thoroughness of handwashing by students?

How easy / difficult is it for you to wash your hands in schools?

What stops you or others doing this? (availability of hand washing facilities, bullying in bathrooms)

What could help you do this? (verbal and written reminders, handwashing incorporated into timetabling, additional facilities, provision of hand sanitisers)

What times in the day is it easier/more difficult to do this?

- **Test and trace** (need to know more about how exactly it is being implemented)

Your school is participating in testing for COVID-19 and tracing students and families. What are your views on this? (is it important understand how the virus spreads among young people and in schools to help control outbreaks?)

Do you have any concerns about participating in this study?

How do you feel about the test itself (saliva sampling (taken yourself) once a month for 6 months, and reporting symptoms to school. Infection will be linked to home postcode & linked to NHS data to understand who is getting the virus & where)

How do you feel about the reporting of the results (Only positive test results will be sent out (no news = good news) – would you have any concerns about this?)

If there is a confirmed case from school testing – all members of household/contacts will be asked to provide weekly samples for 4 weeks – how do you feel about this? Any problems with this? Why? How to do this without causing issues/concerns?

Positive results will lead to households having to self-isolate. What would that mean for your family?

- **Attitudes and feelings towards vaccine**

University of BRISTOL

CoMMinS
COVID-19 Mapping and Mitigation in Schools

School life during COVID 19

TOPIC GUIDE FOR SCHOOL STAFF

Research Objectives

**COVID-19 physical mitigation measures**<br>• Provide details on school context and how COVID-19 mitigation measures have been implemented (facilitators and barriers to their adherence)<br>• Collect evidence on views of sense of physical and emotional safety including attitudes to the measures<br>• Explore views on test, trace and isolate policies and implementation in school<br>**Mental health and wellbeing**<br>• Identify schools' plans for supporting the mental health and wellbeing of students and staff<br>• Identify facilitators, barriers and additional support needed to ensure wellbeing, including those most at risk<br>• Explore how strategies and activities for wellbeing can be enhanced, adapted<br>• Identify best practice and recommendations that could be spread

A. **Setting the scene**

A. Check demographic details and confirm role in school, years of experience teaching etc.
   - (if needed i.e. not completed EofI)

B. Thanks for agreeing to take part. First of all I would like you to tell us about your role in school

C. Tell us a little about your school and students

D. What motivated you (your school) to participate in the overall CoMMinS study?

B. **<u>COVID-19 physical mitigation measures</u>**

A. What guidance and support have you had regarding COVID-19 safety in your school?
   - *Prompt: Anything from Bristol City Council, Department of Education, local public health team?*
   - *Prompt: Any relevant school policy updates?*
   - *Prompt: Designated COVID-19 key person?*

B. What physical mitigation measures have been put in place in your school?

- *Prompt: Social distancing, one-way systems, hand hygiene, ventilation, staggered start times, bubbles?*

C. How has the school responded to the COVID-19 pandemic over the last year? Has the response differed in March, September, November, January?
   - *Prompt: Have these changed as the guidelines changed?*

D. What have been the main challenges?
   - *Prompts: Facilitators and barriers to adherence?*
   - *Prompt: Have the students (age differences/certain groups impact?)/ staff found it difficult to adhere to these measures? Why?*
   - *Prompt: What are your thoughts on using alternative spaces to reduce bubble sizes? (feasible?)*

E. Concerns regarding the physical mitigation measures? How have/could you dealt deal with these concerns?

F. Has anything gone well? Anything that has facilitated implementation and compliance with physical mitigation measures?
   - *Prompt: What has been easy to implement? Prompt: What would be easy to continue with?*

G. What additional support is needed to ensure adherence to COVID-19 mitigation measures?

H. How have these measures been planned, organised and implemented?
   - *Prompt: Staff meetings, inset days, decided and disseminated from Academy chain board, policy updates, with school community?*

I. How do you think the test, trace and isolate system is working?
   - *Prompt: How was it implemented in your school?*
   - *Prompt: Have you had any feedback, concerns from staff, school community about track and trace?*

J. How has communication been managed – among school staff and with parents and students?
   - *Prompt: Is the school community satisfied with communication?*
   - *Prompt: Use of online platforms to communicate?*

K. We are interested in the impact of the mitigation measures on the mental health and wellbeing of your staff and students, can you share any thoughts on this?
   - *Prompt: what issues have arisen directly as a result of these?*
   - *Prompt: activities normally in place for wellbeing that have stopped?*
   - *Prompt: impact of measures on staff? Additional pressures?*
   - *Prompt: impact of measures on students? Key issues?*
   - *Prompt: do the school community feel safe? Physically and emotionally?*

Mental health and wellbeing: Impact on students, staff and school community

STUDENTS

A. What effect do you think the COVID-19 pandemic has had on the mental health and wellbeing of students in your school?

- *Prompt: What about academic impact, exams, future?*
- *Prompt: Social and emotional development?*
- *Prompt: Safeguarding?*
- *Prompt: Inequalities?*

C. What do you think has influenced the wellbeing of students the most?
D. What measure has had the biggest impact on MH in students and staff?

E. Most frequent mental health issues that have worsened/arisen?

- *Prompt: Impact on loneliness, social media use, self-harm, sleep etc.*
- *Prompt: Could these be lasting effects?*

B. Have there been particular groups of young people more affected than others?

- *Prompt: Differences between year groups, other groups? (key workers/vulnerable, ethnic groups, exam years, transition e.g. Year 7?)*
- *Prompt: Can you say how and to what extent?*
- *Prompt: Could you say why they may be more affected?*

STAFF

A. How do you think school staff have been affected?

- *Prompt: Have some been more affected than others? Can you say how and to what extent?*

B. What measures do you think have the biggest impact e.g. managing home learning, safeguarding? *(workload, covering staff absence, illness and loss)*

- *Prompt: Impact on staff relationships and identity?*

C. How have you been affected personally as a senior leader in your school? (if relevant)

- *Prompt: What has the impact been on your role?*
- *Prompt: Impact on relationships with staff, students and families?*

SCHOOL COMMUNITY

A. How would you describe the school community's wellbeing?

- *Prompt: What are your biggest concerns regarding the school community's wellbeing?*
- *Prompt: Access to services, resources, long-term impact?*
- *Prompt: How do they monitor the wellbeing of the school community?*

B. What guidance and support have you had regarding the promotion of mental wellbeing for:

- *Students? Parents/families? Staff? Whole school community?*
- *Prompt: Guidance documents, websites/ access to support workers/ links with organisations?*
- *Prompt: How have you disseminated this support?*

- *Prompt: If any what do you think is most informative? If none, where do you think this guidance should come from?*

C. What strategies/activities have you implemented to support mental health and wellbeing since the first lockdown in March 2020?

D. What plans did you make to support the mental wellbeing of students and staff?
   - *Prompt: How are they working?*
   - *Prompt: How have they changed over time?*
   - *Prompt: How do you think students are interacting with the strategies implemented?*
   - *Prompt: Staff engagement? Access to staff?*

What if anything may help student and staff wellbeing?

A. What do schools need? (and facilitators and barriers)
   - *Prompt: What could be done better and by who?*
   - *Prompt: What additional support is needed?*

B. Can you give any examples of best practice to support mental health and wellbeing during COVID-19 in your school? (or from things seen/read elsewhere, creative ideas)

New Questions Staff:

- Have you experienced a bereavement during COVID- or as a result of COVID-19 in the family or school community?
- (Prompt: have students reported any significant bereavements to the school)
- Are there specific implications on safeguarding as a result covid and the impact on staff and have these or will these be addressed. How can the be addressed.
- How has covid impacted retention and recruitment / training of staff?
- How you received any training on teaching during the pandemic or recovery curriculum?
- Have you received any MH and well-training during the pandemic?
- Have you received any safeguarding training during the pandemic?
- (**Prompts**: if yes no/do you know the procedure of reporting safeguarding)
- (**Prompts**: do you record on CPOMs-safeguarding system for schools)
- What have you learned about MH and WB in your training?
- What have you learned about MH and WB throughout the pandemic?
